# Claims of the Public upon Professional Benevolence

**Published:** 1847-08

**Authors:** 


### PART IV.—SELECTIONS.

1. *Claims of the Public upon Professional Benevolence.*—A woman in labor, at Birkenhead, England, applied to two medical practitioners to attend her, who declined undertaking the case gratuitously. One of them subsequently consented to act on promise of payment, and on finding that the case was a difficult one, procured also the assistance of another practitioner, by whom the woman was delivered. She died in a few days. A coroner's jury was convened, who expressed "their regret that more prompt and efficient aid was not rendered by the medical men in attendance on the deceased;" at the same time "they begged to thank Mr. S. for his prompt attention to the deceased when called upon." The gentleman who consented to take charge of the case, called in the evening and again in the morning after delivery but not receiving the promised payment, declined further attendance. Our respected cotemporary, the Prov. Med. and Surg. Journal, (Wedesday, April 7th, 1847,) has some remarks upon the verdict in this case so just, and as we think, so necessary to be held in remembrance as guides both to the practitioner and the public, that we feel bound to reproduce them.—*Annalist.*

"The poor woman, it seems, fell a victim to the consequences of a natural process, requiring the assistance of a professional man, not on the moment, be it observed, but of the necessity for which she and her friends must have been cognizant for months, and yet no provision for obtaining such assistance is made beforehand, and the medical man, whose time is his estate, and his exercise of his professional calling his means of subsistance, is expected to *give* that relief immediately when demanded, and censured by the public for non-compliance. We should be glad to know on what grounds, as far as the public is concerned, he is answerable to the demand on his time, or amenable to the reproof so liberally bestowed? Why is he to be publicly reproved for not bestowing his guineas in the exercise of his calling, any more than any individual juryman, or other person, who might have the means at his disposal, for not himself handing out the fee, and thus bespeaking and requiting the service performed Let the circumstances of the case be changed, and others, unhappily in this day, of greater frequency, substituted, and let the inquest be supposed for one moment, to have been into the cause of death of an individual, or a family, who may unfortunately have fallen a sacrifice to want of the common necessaries of life; would the jury venture to record a regret that more prompt and efficient aid had not been rendered by any baker or other provision dealer, at whose hands relief might have been sought? or would the account have been headed in a public newspaper, " Alleged misconduct of the provision merchants of Birkenhead," or of Liverpool, or of any other place in which the unhappy event might have occurred? Would they not rather have lamented that the parochial or borough authorities—that is, the public authorities—the authorities to the support of which the public contributes, and over the efficiency of which they have the right of control, had not been more vigilant in the discharge of the duties assigned them? We deny altogether the right of juries thus to censure the conduct of private individuals, or to dictate the scale on which their benevolence should be exercised, and we greatly question whether there was one individual in the court then present, the medical men excepted, who would not have resented the being called out of bed to give hours of attendance in a case requiring assistance, for which previous provision should have been made, to say nothing of contributing his guinea, or the equivalent of it on the spot. It is true we find them liberal of their thanks to Mr. Stevenson, for the gratuitous exercise of his professional skill and attention on the occasion, but no expression of willingness to contribute any portion of their substance towards sharing with Mr. Stevenson the work of benevolence to which he had been devoting his valuable time and professional skill.

Once more we are desirous that the tenor and intent of the preceding observations should not be misunderstood. God forbid that the time should ever arrive when the medical profession is not open, heart and hand, to the call of the afflicted, and willing to afford with genuine disinterestedness, all the aid and consolation—professional or otherwise—in their power, to any one who may ask it; but let not the public claim *as a right* from medical practitioners that professional assistance which it is the duty of the public themselves to see shall be provided for those who stand in need of it, and for which it is equally the duty of the public to see that the professional man who renders it is equitably and liberally remunerated."

---

2. *The Connecticut Legislature,* we are told, have passed a resolution declaring that Dr. Horace Wells, of Hartford, was the original discoverer of the inhalation of ether for surgical purposes. In a paper in the Boston Jour., Jan. 30th, entitled "A Review of Dr. M. Gay's Statement of Dr. C. T. Jackson's Claims, &c." by J. B. S. Jackson, M.D., of Boston, it is acknowledged that Dr. W., in Nov., 1844, did use the sulph.

